# Impact of personalized three-dimensional (3D) printed pelvicalyceal system models on patient information in percutaneous nephrolithotripsy surgery: a pilot study

**DOI:** 10.1590/S1677-5538.IBJU.2016.0441

**Published:** 2017

**Authors:** Hasan Anil Atalay, H. Lütfi Canat, Volkan Ülker, İlter Alkan, Ünsal Özkuvanci, Fatih Altunrende

**Affiliations:** 1 Department of Urology, Okmeydani Training and Research Hospital, Sisli, Istanbul, Turkey;; 2 Department of Urology, İzmir Tepecik Training and Research Hospital, Izmir, Istanbul, Turkey;; 3 Department of Urology, Istanbul Medical School, Çapa, Istanbul, Turkey

**Keywords:** Calculi, Technology, Lithotripsy

## Abstract

**Objective:**

To investigate the impact of personalized three dimensional (3D) printed pelvicalyceal system models on patient information before percutaneous nephrolithotripsy surgery.

**Material and Methods:**

Patients with unilateral complex renal stones with indicatation of percutaneous nephrolithotripsy surgery were selected. Usable data of patients were obtained from CT scans as Digital Imaging and Communications in Medicine (DICOM) format. Mimics software version 16.0 (Materialise, Belgium) was used for segmentation and extraction of pelvicalyceal systems. DICOM format were converted to Stereolithography file format. Finally, fused deposition modeling was used to create plasticine 3D models of pelvicalyceal systems. A questionnaire was designed for patients to assess personalized 3D models effect on patient’s understanding their conditions before percutaneous nephrolithotripsy surgery (PCNL). The day before surgery, each patient was seen by a urologist to deliver information about surgery. Questionnaire forms were asked to patients complete before and after presentation of 3D models and the results of the questions were compared.

**Results:**

Five patient’s anatomically accurate models of the human renal collecting system were successfully generated. After the 3D printed model presentation, patients demonstrated an improvement in their understanding of basic kidney anatomy by 60% (p=0.017), kidney stone position by 50% (p=0.02), the planned surgical procedure by 60% (p=0.017), and understanding the complications related to the surgery by 64% (p=0.015). In addition, overall satisfaction of conservation improvement was 50% (p=0.02).

**Conclusion:**

Generating kidney models of PCSs using 3D printing technology is feasible, and understandings of the disease and the surgical procedure from patients were well appreciated with this novel technology.

## INTRODUCTION

Three-dimensional (3D) printing is a new technology that has developed rapidly in recent years. This new approach holds significant benefits for medical procedures such as maxillofacial reconstruction (1). It has also been used for forensics, orthopedics, and rare complex interventions (2). Non-biological 3D printing models are being applied in the Urology field for planning surgeries, resident education, and patient information (3, 4).

Percutaneous nephrolithotripsy (PCNL) is a standard, safe, and efficient method for treating renal stones larger than 2cm in size (5). Access through an appropriate calyx and knowledge of the complex 3D internal anatomy is essential for a successful PCNL. In the past, intravenous urography (IVU) and ultrasonography were used to analyze pelvicalyceal systems (PCSs) and stone anatomy. With advances in CT technology (e.g., rapid spiral acquisition and reconstruction software), it is now possible to provide accurately-reconstructed 3D images of the PCS that have been used successfully to facilitate successful PCNLs (6).

There is now a growing interest in providing information to support patient’s participation in choosing treatments and deciding on strategies for managing their health problems (7). Information materials are no substitute for good verbal discussions, but consultations are usually short and plenty of evidence exists that patients do not receive the information they want and need (8). For this reason, the ability to generate 3D models from patient data is allowing physicians to inform patients in ways never seen before (9).

In this study, our aim was to assess whether personalized 3D printed models of PCSs can improve patient’s understanding of their conditions before PCNL surgeries.

## MATERIAL AND METHODS

### Creating 3D Printed Models from Medical Imaging

Creating 3D models from medical imaging data is a multi-step process. First, usable data must be obtained from CT scans, magnetic resonance images, or ultrasound images. In our hospital (Okmeydani Teaching and Research Hospital), we used CT (Toshiba Alexion™ multislice CT) scan data from five patients. Data from CT scans in Digital Imaging and Communications in Medicine (DICOM) format are important because low-resolution images can result in inaccurate models (10).

Second, segmentation, or extraction and isolation of the area of interest, of the data must be performed. We sent our DICOM-formatted data to a bioengineer (Biotechnica Engineering Co Ltd, Istanbul) for segmentation. Numerous software programs are available for use with DICOM datasets. In our case, Mimics software version 16.0 (Materialise, Belgium) was used.

Finally, data must be saved in a file format recognized by the 3D printer software. The most commonly used format is the Stereolithography (stl) file format.

### 3D Printing

Recent advances in 3D printing technology have produced new processes that allow the use of a variety of materials factors. Acrylonitrile butadiene styrene (ABS) has been used for creating 3D models of PCSs. The most important mechanical properties of ABS are impact resistance, toughness, high radiodensity, and low cost. We used fused deposition modeling, an inexpensive technology popular with consumers. These printers use a polymer filament that is heated to a liquid state in a printer head and deposited in predefined locations corresponding to the model shape (Stratasys Inc.) (11).

### Evolution of Personalized 3D Printed Models from Patients

A survey questionnaire with open ended questions of ordinal 10-point rating scales (1-poor/fair/good, 10-very good/excellent) was given to patients which consisted of 3 components: a) overall satisfaction of conversation (1 item), b) model assistance in understanding the disease and procedure (3 items) and c) understanding the complications related to the surgery (1 item). The day before surgery, each patient was seen by a urologist, the IVU images and CT scans were used as a teaching aid to deliver information about surgery and the disease. After the conversation, the questionnaire form asked patients to complete and then 3D printed models were presented to patients and they completed the questionnaire form again. Results of the questions were compared before and after the presentation of the 3D models.

### Statistics

A total of 25 questions were asked to patients. Median total scores of responses for each category, before and after 3D printed model presentation, was compared (Wilcoxon test). Statistical analyses were performed using SPSS Statistics version 21.0 (IBM Corporation, Armonk, NY, USA)

## RESULTS

**Figure 1:**
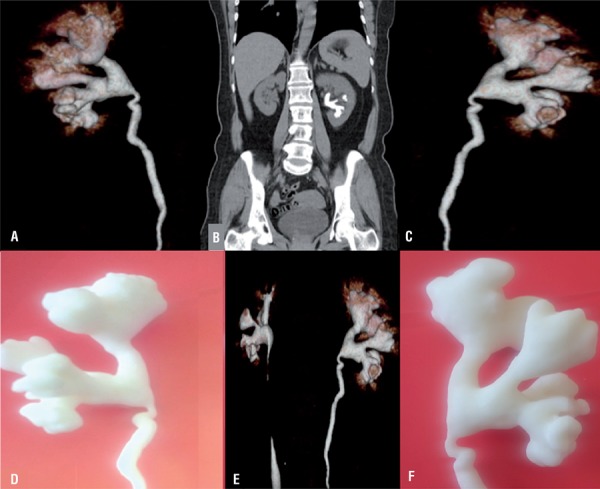
Patient 1 A) Posterior view of collecting system, B) Coronal view of collecting system, C) Anterior view of collecting system, D) Posterior view of 3D printed model, E) Posterior-lateral view of 3D printed model, F) Anterior view of 3D printed model.

**Figure 2:**
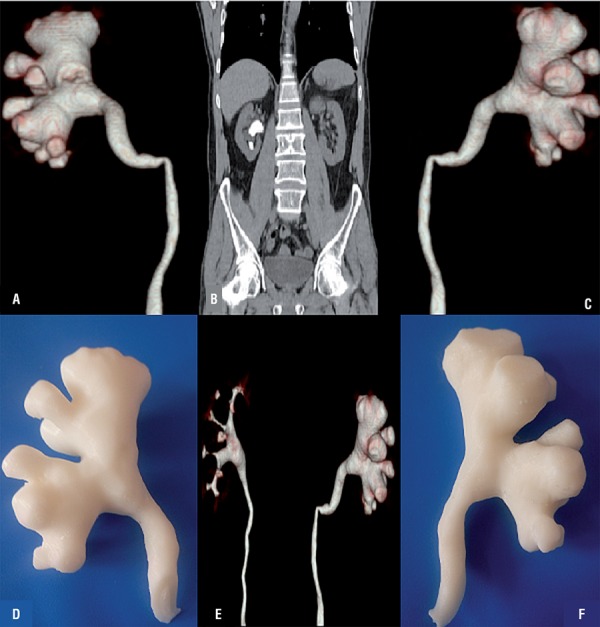
Patient 2 A) Posterior view of collecting system, B) Coronal view of collecting system, C) Anterior view of collecting system, D) Posterior view of 3D printed model, E) Posterior-lateral view of 3D printed model, F) Anterior view of 3D printed model.

From June 2015 to January 2016, 5 patients with a unilateral staghorn renal stone and clinical indication of percutaneous nephrolithotomy were selected. Our first aim was to successfully create a 3D model of the pelvicalyceal systems. After two attempts we successfully generated anatomically accurate human renal collecting system. Overall collecting systems were clearly presented with regards to the virtual reconstructions. Fused deposition modeling with Acrylonitrile butadiene styrene (ABS) was used to generate anatomically-correct size and shape renal collecting system. The consumable costs of the model are low, at around 100$, and the print time for the 3D model is approximately 2 hours (Figures 1 and 2).

Patients demonstrated an improvement in their understanding of basic kidney anatomy, planned surgical procedure and understanding the complications related to the surgery after viewing their personal 3D kidney models. After the 3D printed model presentation, it was found that the mean improvement rate of total scores was higher. Patients demonstrated an improvement in their understanding of basic kidney anatomy by 60% (p=0.017), kidney stone position by 50% (p=0.02), the planned surgical procedure by 60 % (p=0.017), and understanding the complications related to the surgery by 64% (p=0.015). In addition, overall satisfaction of conservation improvement was 50% (p=0.02).

## DISCUSSION

Accurate information which patients find useful has the potential to enhance the quality and appropriateness of health care. However, using only 2D images makes it very difficult for surgeons to inform their patients about the surgery and the complications. This is why new modalities have been developed for patient information (12). PCNL is an effective method but various factors have a negative impact on the success rate and complications of PCNL surgery. Several studies have revealed that increasing stone burden and multiple tracks correlate with decreased stone-free rate and increased bleeding (13). Although staghorn stones required multiple tracks to have a stone free status. The ultimate goal would be complete stone clearance with no complications.

In the present study, we have created 5 physical, patient-specific, anatomically identical human renal collecting system 3D models before operative intervention, based on CT imaging of patients with unilateral staghorn renal stones. 3D models of the PCS can provide not only necessary images but are also feasible in planning collecting system access for planning PCNL surgery in complex staghorn renal stones.

Printed models have been used for preoperative planning in complex orthopedic and craniofacial procedures and neurosurgery (14, 15). There have been a few reports about bio-modeling for planning endourologic procedures and usefulness as an education tool for patients (16). While creating a 3D model, CT scan slice thickness has to be 5mm or up to 3mm because low resolution images can result in discrepancy between the generated model and actual anatomy (17). But the increased radiation dose delivered with CT scans thinner then 5mm, is a cause of concern. The high-dose could increase 75 per cent of the radiation exposure compared to low-dose CT, according to the literature reports (18, 19).

Generating anatomically identical 3D models of renal collecting systems allows surgeons and patients to interact with the renal unit in a tangible way than using conventional images. In this study, it has shown that this interaction with models is an effective educational tool for patients resulting in an improvement over conventional imaging. With the 3D models, patients were better able to understand the renal anatomy and renal physiology, and the complications related to the surgery compared to CT scans and IVU images.

This study has shown that, physician and patient communication were improved after presentation of personalized physical 3D models. 3D physical models are valuable for patient satisfaction of conservation with a 50% improvement. Despite given detailed information with IVU and CT images about planned surgery and complications that may occur, patient’s initial reference level of understanding was low. It is very difficult for patients to understand IVU and CT images and it can also be difficult for physicians to inform patients about the surgery and associated complications. After the presentation of 3D models, we witnessed how they helped patients raise and ask their own questions, enhancing their understanding. Improving patient education by the use of personalized 3D printed models appears to be a promising way to efficiently enhance the quality of personal exchange between a patient and his surgeon and influence overall patient satisfaction. In addition, patient education level is effective on the disclosure process.

Moreover, using this kind of personalized 3D models not only for patient counseling but also for students, residents and fellow’s surgical teaching could help achieve better cost-effectiveness. Indeed, such models, in making easier 3D anatomical understanding, may certainly be useful tools to enhance surgical strategy discussion and improve preoperative planning.

## CONCLUSIONS

Generating kidney models of pelvicalyceal systems with 3D printing technology is feasible; understanding of the disease and the surgical procedure from patients were well appreciated with this novel technology.

## ABBREVIATIONS

3D: Three dimensional
CT: Computed tomography
IVU: Intravenous urography
PCNL: Percutaneous nephrolithotripsy
PCS: Pelvicalyceal systems
DICOM: Digital Imaging and Communications in Medicine
Q: Questions
